# Advanced e-Call Support Based on Non-Intrusive Driver Condition Monitoring for Connected and Autonomous Vehicles

**DOI:** 10.3390/s21248272

**Published:** 2021-12-10

**Authors:** Marius Minea, Cătălin Marian Dumitrescu, Ilona Mădălina Costea

**Affiliations:** Department Telematics and Electronics for Transports, University “Politehnica” of Bucharest, 060042 Bucharest, Romania; ilona.costea@upb.ro

**Keywords:** automated vehicles, connected vehicles, driver condition monitoring, safety stopping, emergency automated call, wireless sensor network

## Abstract

Background: The growth of the number of vehicles in traffic has led to an exponential increase in the number of road accidents with many negative consequences, such as loss of lives and pollution. Methods: This article focuses on using a new technology in automotive electronics by equipping a semi-autonomous vehicle with a complex sensor structure that is able to provide centralized information regarding the physiological signals (Electro encephalogram—EEG, electrocardiogram—ECG) of the driver/passengers and their location along with indoor temperature changes, employing the Internet of Things (IoT) technology. Thus, transforming the vehicle into a mobile sensor connected to the internet will help highlight and create a new perspective on the cognitive and physiological conditions of passengers, which is useful for specific applications, such as health management and a more effective intervention in case of road accidents. These sensor structures mounted in vehicles will allow for a higher detection rate of potential dangers in real time. The approach uses detection, recording, and transmission of relevant health information in the event of an incident as support for e-Call or other emergency services, including telemedicine. Results: The novelty of the research is based on the design of specialized non-invasive sensors for the acquisition of EEG and ECG signals installed in the headrest and backrest of car seats, on the algorithms used for data analysis and fusion, but also on the implementation of an IoT temperature measurement system in several points that simultaneously uses sensors based on MEMS technology. The solution can also be integrated with an e-Call system for telemedicine emergency assistance. Conclusion: The research presents both positive and negative results of field experiments, with possible further developments. In this context, the solution has been developed based on state-of-the-art technical devices, methods, and technologies for monitoring vital functions of the driver/passengers (degree of fatigue, cognitive state, heart rate, blood pressure). The purpose is to reduce the risk of accidents for semi-autonomous vehicles and to also monitor the condition of passengers in the case of autonomous vehicles for providing first aid in a timely manner. Reported abnormal values of vital parameters (critical situations) will allow interveneing in a timely manner, saving the patient’s life, with the support of the e-Call system.

## 1. Introduction

The trend now in worldwide automotive transportation is the rapid changing of propulsion solutions from fossil fuel to electric stored energy or to hydrogen-based fuel cells. Both solutions have advantages and drawbacks. Both need a dedicated infrastructure construction, considering the distance between power supplying facilities, or hydrogen re-charging stations. Neither of these two solutions would be beneficial for the environment if the supplied energy would still be produced by classical means (burning of fossil hydrocarbons or coal). For such a complex investment and economical change to be effective, only the use of green (renewable) energy is allowed. Moreover, the new developments now point towards connected and autonomous vehicles, despite the fact that the road network is not yet prepared for a mixture between non- and autonomous vehicles. Therefore, we consider that firstly connecting vehicles and sharing relevant safety and dynamic information might prove crucial for traffic development in safer conditions. In the conditions of mixed traffic, detecting dynamics and estimating trajectories is essential for reducing the accident rate. However, let us not forget that many of the traffic accidents have their origin in bad driving or driving in a non-compatible health state (drunk people or with other health conditions incompatible with driving a vehicle, such as the consumption of illegal substances). Therefore, the development of a solution with minimal investigation of driving conditions and/or passenger monitoring will be beneficial for reducing the chances of an incident and helping emergency services with some previously collected medical information in case the accident has already happened.

Between the elements that could affect the consciousness state of the driver or the ability of the person in charge to take control of an automated vehicle in case of necessity, the following conditions must be considered:

- Cabin noise level/pattern and ambience that may affect the attention of the vehicle driver.
- Temperature control in the cabin.
- Atmosphere control in the cabin, including gas concentrations of CO, NOx, PM, etc.
- Effects of vibration patterns (frequency, amplitude, direction).
- Ambient light and direct solar light influence on road visibility or on the attention the driver pays to its actions.
- For electric cars, effects of long-term exposure to different electromagnetic fields.
- On-site or remote detection on consciousness state and attention in driving/surveillance of autonomous vehicle driving.

Of course, this way of monitoring the driving cabin conditions is complex and involves installing many sensors, in exchange having negative effects on the complexity and reliability of the vehicle. However, because all these conditions are meant to be monitored for maintaining a good state of consciousness and attention of the driver, a simpler way would be the direct monitoring of these elements. In a future environment of connected semi- or autonomous vehicles, one of the traffic safety conditions would be the ability of a driver to take control of the vehicle and respond to complex situations, if necessary. Therefore, a constant monitoring of the driver condition should be included, along with vehicle dynamics monitoring, among the safety conditions that allow the movement of the vehicle. Otherwise, if any abnormal situation is detected, the vehicle should be able to come to a complete stop in a safe manner. The early detection of driver fatigue caused by sleepiness is a key technology able to decrease the rate of fatal accidents. In a connected vehicles environment, this detected information should also be used for announcing the neighboring vehicles about the immediate stopping of the vehicle of which the driver is no longer able to ensure safe driving conditions. Moreover, if a collision occurs, pre-recorded information regarding the number of passengers or the driver state before the collision could serve as primary information for medical help.

The paper exposes the research and experimentation activity during a prototype. To begin with, the exact requirements of the system for monitoring the interior of a vehicle are determined. The design and implementation of the systems follows, with the end detailing the resulting devices, the data collected from the prototype mounted on the test vehicle, and a procedure for using them. Nowadays, people spend a lot of time in a car, mostly in traffic jams. Sensors based on MEMS (Micro-Electro-Mechanical Systems) technology are small in size and suitable for multi-sensorial integration. Theoretical and practical aspects were studied, such as methods, techniques, and systems already existing today, in order to analyze the biomedical signals, temperature measurement techniques, gesture detection, IoT device management and the use of MEMS sensors. Several prototypes have been developed with the aim of solving problems of interest in academia and research, with a possible wide applicability in the automotive industry, where the emphasis is put on the development of non-contact sensors for the acquisition of biomedical EEG and ECG signals. The interface for viewing and managing data from multiple IoT devices has been designed.

In order to solve problems of interest in academia and research, several prototypes have been developed, with a possible wide applicability in the automotive industry, where the emphasis is on the development of contactless sensors. The sensors were installed in the headrest and the back of the driver’s seat, without the human subject and without the subject being embarrassed in any way. EEG and ECG sensors can detect and collect biomedical signals from a distance of up to 10 cm. Additionally, by analyzing brain waves in addition to highlighting the state of drowsiness that may occur for the driver, the EEG module presented in the article is also able to detect the cognitive states of the driver/passengers, such as relaxation, emotion, stress and inattention. An interface for observing and managing data on multiple IoT devices has also been designed. The test results showed a detection rate for the analysis of cognitive states of 96.75%.

The rest of the paper is structured as follows: Section 2 presents a literature survey; Section 3 presents the architecture of the IoT model for the integration of sensors for the acquisition of biomedical signals, temperature, presence, algorithms for analysis and detection of cognitive states, ECG parameters and sensors for detecting overheating of vehicle components, as well as the results obtained by the new method proposed together with the algorithms used in Section 4. In addition, in Section 4, a comparison is made between the proposed method for determining EEG cognitive states and some relevant articles dealing with the same topic.

We consider that the present research might be helpful in the enhancement of safety data exchange. The advantage of this proposal is that it integrates different technologies in a non-intrusive package, allowing for a permanent observation of the conscious and health-related state for the person occupying the driver’s seat, with the possibility of extending it to other vehicles’ occupants. Moreover, the application is oriented towards a direct support for e-health services, improving the efficiency in saving human lives in road accidents.

## 2. Literature Survey. Related Work

Intensive research has been pointed in this direction. Luca Salvati et al. [1] propose an algorithm to detect the sleepiness state of a driver based on the measurement of pulse rate variability generated by heartbeat. Then, the proposed method is validated via an objective indicator (time-mediated percentage of eye closure—named PERCLOS) [2]. The authors carried an experiment with three persons in a car exhibiting different conditions, for which pulse rate had been monitored to detect autonomic nervous system (ANS) state by the heart rate variability (HRV). In the same area of activities, studies on the possibilities to detect driver state have been heading in two directions:

- Measurement of psychological-related state signals, such as electroencephalogram (EEG) [3,4], eye movement and closure rate (EMCR) [5], longitudinal (Gripps modified) model [6], and AI-based analysis of face characteristics or AI-based analysis of posture and behavior (position of sitting, head position, etc.).
- Analysis of driving behavior and patterns with information collected from the vehicle’s sensors and safety-related systems, such as lane keeping assistant (LKA) [7], steering wheel movements, acceleration, and other dynamic parameters. Of course, this second approach is available only for non- or semi-automated vehicles that are driving.

In the direction of a psychological parameter assessment, Dong Eun Lee et al. present [8] a method for driver-gaze tracking based on a fuzzy-system method for detecting a driver’s pupil and corneal specular reflection in a cabin of a vehicle. They consider two features, which are passed through a fuzzy system, i.e., the symmetrical characteristics of face and facial feature points to determine the status of a driver’s head rotation. The method suffers the disadvantage of employing video analysis, which might be negatively influenced by the ambient lighting. J. Chang et al. [9] extend the research and propose a context-aware service system that is able to ensure a configurable architecture for the design and implementation of a specific health service system designed to increase safety in traffic. The solution is designed to detect the driver’s health status and provide helpful services to the driver. In addition, Lee B. et al. propose employing remote physiological monitoring using Bluetooth to measure main parameters of consciousness state, such as electroencephalogram (EEG) and respiration signals of a driver in the time and frequency domains [10]. In order to reduce the stress of connected sensors or measurement techniques that might disturb the driver from his main tasks, Barbusiak et al. [11] propose employing a smart steering wheel to record and analyze the movement patterns performed by the driver while driving. The developed intelligent steering wheel is usable in applications designed for monitoring the driver’s state of health, drowsiness, or driving style. The disadvantage of that method is that it only monitors a single health parameter. Other authors [12] develop a solution for evaluating the cognitive workload for a professional driver health examination to monitoring the mental state of people carrying out jobs of high responsibility, such as train drivers, pilots, or airline traffic dispatchers. They employ offline evaluation, with data acquisition based on registering the EEG signal of the person performing arithmetical tasks divided into six intervals of advancement. The analysis then includes preprocessing, feature extraction, and selection. The final phase executes multiclass classification using several models. The main disadvantage of this methodology is that it is not applicable while driving, but it could be useful in driving schools or certification procedures. In [13], Wang J. et al. offer a guide to currently proposed sensor systems for in-vehicle bio signal monitoring. Additionally, technologies such as radar and capacitive measurements are used [14] for unobtrusive monitoring of drivers’ physiological parameters. Here, the authors also put an accent on investigating the influence of vibrations and other disturbances that may occur during the vehicle’s travel. This solution has the disadvantage that it employs radiation of the subject with a high frequency electromagnetic field. Internet of things (IoT) may also be used to connect various measurement devices used for the assessment of the consciousness state of a driver, and such work is proposed in [15]. The authors developed a solution for minimizing road accident risk by integrating technologies employing mobile devices (phones) for ensuring medical assistance (mHealth technologies) with a vehicular information system (VIS) using wireless body area network sensors and devices. The method employs sensors directly in contact with the subject. They also propose a secure environment, considering the data privacy. Janis Dröge et al. [16] perform research on measuring the exposure to particulate matter (PM) in a car cabin employing a mobile aerosol spectrometer. The atmosphere of the driving cabin is also important for the health state of its occupants. In a similar scenario of measurements, the authors of [17] perform experimental research on monitoring the carbon dioxide (CO_2_) and total volatile organic compounds (TVOC) inside road vehicles with different cabin sizes and with various numbers of occupants.

Various sensors were installed for detecting especially the health state of the driver in different parts of the cabin: the steering wheel, back seat, headrest, rear looking mirror, sun visor, etc. Such an example is given by [18], where the authors use the seatbelt for including RFID low-powered detectors for monitoring seat belt state in buses. This does not represent a direct measurement of passenger state of health but is a good approach for increasing safety in very large passenger vehicles. Pointed towards the same area of driving safety, an interesting application is presented in [19], where Alessandro Leone et al. propose a module for Advanced Driver Assistance System (ADAS) to be used for minimizing the accident frequency when caused by road rage, alerting the driver when a predetermined level of rage is reached. The solution employs facial characteristics analysis and assessment, having the typical disadvantages of using video cameras inside the driving cabin: influence of incoming lighting. Another direction in the research of that domain now has issues due to the expansion of electric vehicles in traffic: the exposure to increasing electromagnetic fields. In this area, the authors of [20] analyze exposure to extremely low frequencies (ELF) and magnetic fields (MF) in electric vehicles, based on the measurement of flux densities and spectral components. They conclude that it is recommended to periodically monitor ELF and MF, especially after repairs of EVs, to avoid long exposures to potentially dangerous magnetic fields and radiofrequency (RF). An evaluation on practical sensors that have the potential to provide reliable monitoring and meaningful feedback to vehicle operators in the diverse conditions of vibrations, temperature, air pressure and lighting in a cabin of a car is shown in [21].

Returning to the detection of medical parameters of a driver, in work [22], the authors offer a solution for a video-based measurement of the driver’s heart rate to prevent road accidents caused by acute heart diseases, and in [23] a solution is described for a video-based driver state monitoring system employing heart rate signals via capacitively coupled and radar sensors. A human activity recognition algorithm is proposed in [24], employing a various palette of sensors. For reducing the motion artifacts in the use of non-intrusive ECG sensors, the authors of [25] propose an interesting approach: the use of two capacitive ECG sensors (cECGs) for determining the ECG, with an additional two cECGs to obtain the information on motion. Fatigue feature extraction, fatigue feature fusion and driver drowsiness detection are used in [26] in a particular model employing convolutional neural networks (CNNs), applied to video streaming from a camera pointed to the driver. Not directly connected with driving cars and detecting sleepiness in the automotive field, but closely related to it, is a study [26] concerning the use of special light glasses in reducing sleepiness. Causes and effects that lead to drowsiness state are analyzed in [27], and the result of this study classifies the first causes of drowsiness as being: exposure to recent stress situations, medication and sleep deprivation. To determine the reduction in attention by analyzing the heart rate of the driver, in [28], features of electrocardiography (ECG) and electroencephalography (EEG) are detected and processed using a support vector machine (SVM) classifier. The conclusion of this study is that monotony in driving is an important factor in inducing an increased drowsiness state. Research in the same direction, but employing the fusion of several optimized indicators, is based on driver physical and driving performance measures taken from the ADAS system of the vehicle is presented in [28]. Mixed traffic between non- and autonomous vehicles is a problem that raises numerous questions regarding legal regulations in case of an accident. This direction of research should not be left behind due to the numerous implications in legal, social, and economic domains that it has. A study [29] deals with this problem and concludes that contributing factors to the crash severity of an AV are not clearly defined. The authors of [30] try to give a safer mode of driving to AVs by proposing a Model Predictive Control (MPC), based on imitation of human behavior in driving. The same analysis on driver behavior is pointed to a junction in the research of Xiamei Wen et al. [31]. Of course, one solution to the dual problem of mixed traffic between autonomous and non-autonomous vehicles would be the data networking between vehicles, as shown in [32,33]. The autonomous vehicle and the driver make an intelligent human–machine–road system. Some new approaches investigate the effects of introducing self-rotating chairs and even investigate the effects in case of unexpected situations, based on several volunteers who participate in this experiment. In work [34], the authors investigated the take-over reaction time, remaining action time, crash, situation awareness and trust in automation. Remaining in the human behavior-imitating automation, the authors of [35] propose a lane changing decision control protocol. Finally, another interesting development of detecting the human behavior when driving is applied in reference [36], where the authors propose a calibration of autonomous driving functions based on automatic emotion recognition. Similar work is presented in works [37,38,39], and other methods for non-intrusive detection of health parameters are also described in [40,41,42].

In conclusion, we must admit that challenges in this direction of research remain: the necessity to non-intrusively detect human conditions in both non- and autonomous vehicles, to insert human-related parameters in data exchanged between connected vehicles in order to improve traffic safety, and to deal with the problem of mixed traffic.

## 3. Materials and Methods

### 3.1. Driver State Monitoring—Proposed Structure of the Onboard System

As presented above, the proposed solution is designated to improve the transition between man-driven vehicles and autonomous ones by introducing a system to permanently monitor the condition of the person sitting in the driver’s place, no matter the vehicle’s degree of automation. The system is also intended to ensure exchange of safety-related information regarding the ability of taking control, driving the vehicle in a connected vehicles environment, and/or to provide useful medical/passenger information in case of an incident.

**Figure 1:**
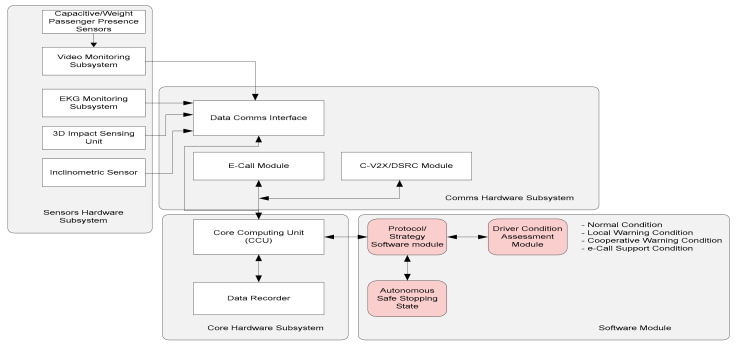
The onboard system architecture.

The internal hardware/software solution is described in the block diagram from Figure 1. The onboard system is composed of the following sub-systems:

- The sensors hardware subsystem (SHM). This functional block enables detection and collection of driver/passengers presence and weight-related data, cabin imaging (with the possibility to include facial detection of drowsiness state of the driver), non-intrusive ECG monitoring of driver, impact direction/intensity measurement, and inclination of the vehicle.
- Communications Hardware Subsystem (CmHS)—includes the modules able to ensure interfacing and communications via GSM/GPRS (if needed for commercial vehicles), e-Call system interfacing, and C-V2X/DSRC communications for a connected vehicles environment.
- Core Hardware Subsystem (CrHS)—ensures the processing of all information and ensures analysis of normal and abnormal conditions of the vehicle and its occupants. It must be enabled with local storage of information in a loop, and in case of an impact detection, with the possibility of storing acquired data of the last few seconds and ability to transmit local imaging, position of vehicle after impact, and other recorded parameters.
- Software Module (SM)—provides the algorithms and software programs designated for data collection, processing, and issuing alarms in case of an abnormal situation detection.

### 3.2. Non-Intrusive Acquisition of Driver State Health Information

The electrocardiogram (ECG) is a frequently used technique for health monitoring, and we may consider it as primary information. Based on ECG and other measured parameters, the algorithms of machine learning will be employed for determining the health state of the driver, with a focus on drowsiness and/or other driving non-compatible states. ECG provides information regarding the heart activity based on the measurement of electrical parameters. It may be used to detect both cardiovascular diseases and heart attacks or other related health parameters that may affect the safety of traffic if applied to a driver.

Detection of attention and/or drowsiness are highlighted by employing an EEG sensor, an analysis of brain waves related to cognitive changes, and by determining abnormal positions of the driver, via a system based on video camera and Deep Learning analysis, to detect abnormal behavior.

The purpose of the small vehicle-integrated medical sensory system is for an early detection of critical conditions and initiate appropriate safety measures, including local warning, cooperative warning, and/or vehicle safe stopping. To safely monitor the driver state (for safety reasons), it is imperative that all sensors do not interfere with the driver’s comfort, attention, or prevent him/her from making movements. Therefore, only non-contact, non-intrusive measurements are taken into consideration. To monitor vital signs as well as the vigilant state, heart activity represents a good source of information, along with breathing rate or the surveillance of facial expressions via an artificial intelligence-based software. For both the mechanical and electrical activities of the heart, there are few methods of non-contact monitoring:

- Capacitive ECG measurement (or cECG).
- Ballistocardiogram-based (BCG) mechanical measurement of heart activity.
- Magnetic impedance measurement of respiratory and heart activities via the determination of rhythmic variations.
- Doppler-radar investigation of heart activity. However, this technique involves a continuous irradiation of the subject, not recommended for long exposures due to its possible secondary effects on health.

**Figure 2:**
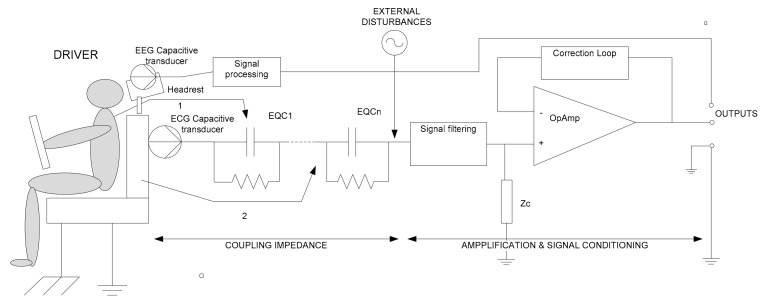
Electronic equivalent circuit of the measurement setup.

Another important aspect should be the integration of sensors in the vehicle’s equipment without perturbing the normal position or activity of the subject or endangering his/her health due to long exposures to contaminants or radiations. From this point of view, the cECG approach seems to be the most appropriate to be installed in the cabin of a road vehicle. The measurement principle is based on the evaluation of the capacity between two electrodes, in between which the human body represents the electrolyte. From the technological point of view, compared to direct contact, resistive measurement-based methods, the evaluation of the signals produced by capacitive measurements need amplification as close as possible to the coupling surface. The electronic equivalent diagram of the measuring principle is presented in Figure 2 below.

Figure 2 presents the equivalent circuit of the capacitive measurement setup installed in the backseat behind the driver. Since there are some insulating layers between the effective body of the driver and the measurement plate (capacitive electrode), their equivalent impedance is represented by a series of inductive-capacitive circuits (LC) circuits, each having the value:

(1) $$Z_{i}=j\omega L_{i}+\frac{1}{j\omega C_{i}}$$

with the total value of:

(2) $$Z_{tot}=\sum_{i=1}^{n}(j\omega L_{i}+\frac{1}{j\omega C_{i}})$$

with the corresponding $L_{i}$ and $C_{i}$ depending on the dielectric properties of the layer that separates the human body from the sensor active plate, i.e., clothes, air, plastic, or textile materials from the backseat, etc. As there is a vehicular environment, a disturbing source of noise should be considered (this can be in the form of inductive currents, capacitive interferences or direct voltage spikes present in the power supplying circuits due to engine ignition, or electric motor inductive effects). To reduce this noisy environment influence, a signal filtering module must be also provided. However, these types of interferences can be significantly reduced if the amplification and signal conditioning module is placed as close as possible to the transducer. In addition, for the linearization of the transducer characteristics, a correction loop might be necessary on the negative amplification input. Due to the relatively limited extent of electrical vehicles, there are no studies regarding the possible influence of the power system of such a type of vehicle on the measurement setup yet. However, according to some research [43], there is also the problem of static electricity accumulation in the measurement setup; therefore, the component *Z_c_* must also be comprised of a discharge impedance in parallel with the input impedance of the amplifier. Modern vehicles include more plastic materials in their components, so there is an increased probability of electric discharges. Causes that might produce static electricity in vehicles may include:

- Vicinity of insulated metal parts, surrounded by plastic or textile materials.
- Unearthed metal ring fasteners.
- Friction between passenger clothes with plastic or textile materials in the cabin, especially in dry weather periods.

**Figure 3:**
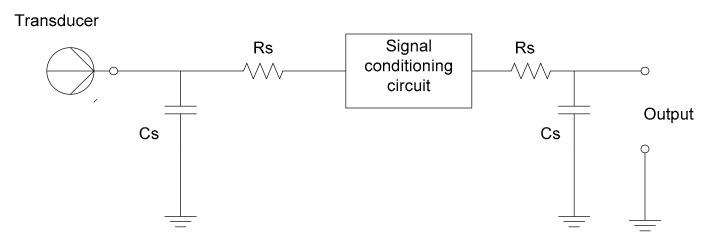
Passive resistive-capacitive (RC) protective circuitry for static electrical discharges.

**Figure 4:**
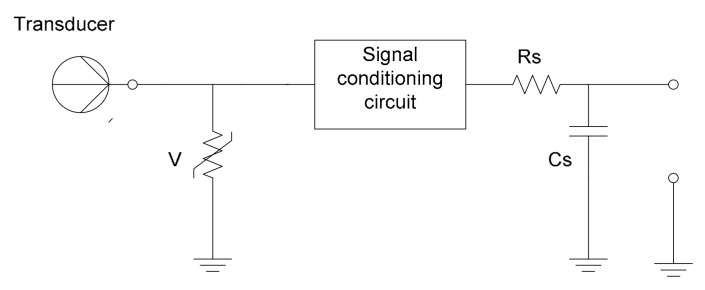
Passive protection using a mixture of a varistor and passive RC protective circuitry.

**Figure 5:**
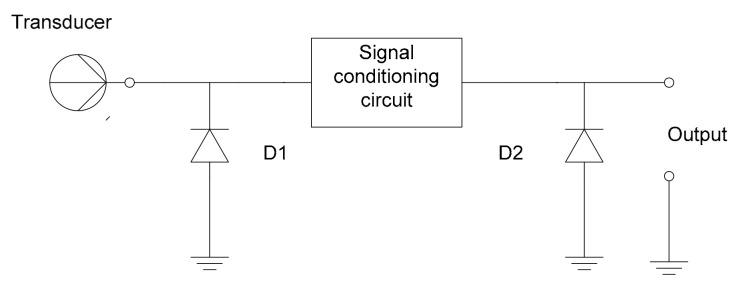
Passive electrostatic discharge (ESD) protection employing diodes.

For permanent protection of the input circuitry of the measurement system against electrostatic discharges (ESD), different solutions might include diodes connecting to the ground, capacitive circuitry, etc., but these measures should be calculated in such a way that they do not affect the high frequencies of the useful signal and, therefore, the accuracy of determining the heart rate and other significant health parameters (Figure 3, Figure 4, Figure 5).

If the measurement system is based on a supervised machine learning solution, then the training should also take into consideration different measurement conditions, such as winter periods, when the driver might wear thicker clothing, or summer, when there is thinner clothing and more humidity due to sweating. The measurement accuracy might vary due to these conditions, and an efficient calibration and fine tuning might be necessary before correctly determining the health parameters of the driver.

### 3.3. Method for Determining Heart Rate (ECG) without Physical Contact with the Subject

In recent years, engineering efforts have focused on developing methods for monitoring heart rate or respiratory rhythm without involving the physical contact of sensors with the subject. The advantages of such an approach are obvious in situations where the investigated subject is the driver of a transport vehicle (car, train, plane), the victim of a fire (experiencing severe burns on the surface of the body), an avalanche or an earthquake, or if the measurement targets military applications—discovering enemies hidden behind walls or assessing the condition of combatants on the battlefield.

The realization of this desideratum (non-contact, remote measurement of vital parameters) has a starting point in our approach by the employment of processing algorithms and a multiresolution analysis based on wavelet decompositions.

**Figure 6:**
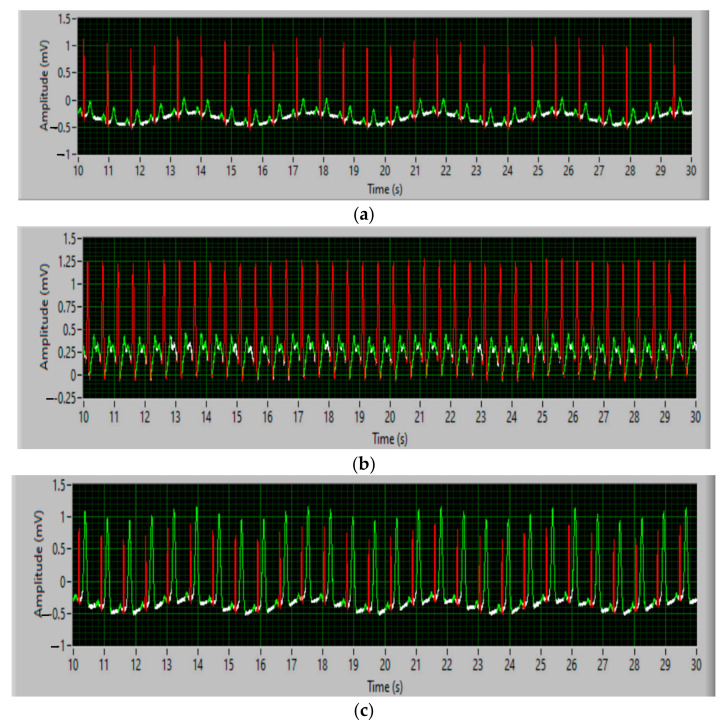
QRS cardio analysis for: (**a**) clinically normal; (**b**) hypotension; (**c**) hypertension.

An ECG signal typical of a cardiac cycle consists of a P-wave, a QRS complex, a T-wave (repolarization), and a U-wave, which is normally invisible in 50–75% of ECGs because it is hidden by waves. T and the new P wave follow the baseline of the electrocardiogram (flat horizontal segments). The ECG signal consists of a series of cardiac cycles, practically a repetition of an ECG wave. For example, the shape of the ECG signal may differ from patient to patient, depending on each person’s health problems. The distribution of characteristics changes depending on the different classes of blood pressure. In this context, through the measurements performed on some patients with heart problems, we found the following:

1. In patients with values within normal heart rate and blood pressure, the shape of the EKG signal is shown in Figure 6a;
2. In patients with heart problems (hypotension), the EKG signal resembles Figure 6b;
3. In patients with heart problems (hypertension), the EKG signal resembles Figure 6c.

#### 3.3.1. Filtering of Technical Interference from the EKG Signal

When acquiring medical signals, there is a risk that the useful information from the subject will be “contaminated” by certain interference induced by the instrumentation amplifier, the signal recording system, or electromagnetic waves from the electronic equipment of a transport vehicle. To these, in many cases, the mains noise (50 or 60 Hz) and its harmonics if the system is powered from the mains are added, with the 50/60 Hz interference being considered a high frequency artifact if we take into account that the spectrum the EKG signal is par excellence a low frequency one (up to 100 Hz) [44,45,46].

#### 3.3.2. Wavelet Transforms (WT) ECG Processing

In the last two decades, one of the topics that enjoyed a high interest is the one specific to signal theory—more precisely, that of the Wavelet transform. With the notable exception of signals whose characteristics cannot be separated from those of the systems that generated them, multi-resolution analysis techniques, especially those that have focused on the use of the Wavelet transform, have always been the focus of many research centers but have also successfully penetrated the most diverse practical applications. Unlike the Fourier transform, about which data are found much faster and in much larger quantities, and which enjoys a much greater popularity so it can be applied much more easily, the Wavelet transform involves obtaining additional information from the signal, which is available in its raw form. In order to overcome the resolution problem, an alternative to the short-term Fourier transform (STFT) is the use of the Wavelet transform.

The wavelet function is the result of a third-band filter and a scaling that aims to halve the bandwidth (problems arise for covering the entire spectrum; to solve this requirement would require an infinite number of levels). The scaling function has the role of filtering the lowest level of the transform and ensures the coverage of the whole spectrum. Multi-resolution analysis allows us to analyze the signal at different frequencies with different resolutions. A Wavelet can provide good temporal resolution at high frequencies and good frequency resolution for low frequency cases. This is not desirable when talking about signal analysis because low frequencies require a slow evolution of the signal, while high ones are found in sudden transitions in the signal, whose “capture” is favored by a good temporal resolution. This represents a “supervised learning” method, in which the basic functions are chosen a priori; detailed information may be found in [47,48,49,50].

This way of sharing the time–frequency plane can be obtained by translating and scaling on the time axis a unique function called the mother wave Ψt:ψs,τ=1sψt−τs, where the scale (*s*) variables and those of positioning on the time axis (τ) are continuous variables. If we try to discretize these variables, we will be able to obtain a discrete version of the parent wave, $\psi_{j,k}\left(t\right)$. It should be noted that it is not the time variable that gives the discretized version of the wavelet, but its other two parameters:

(3) $$\psi_{j,k}\left(t\right)=s_{0}^{\frac{-j}{2}}\psi \left(s_{0}^{-j}t-{k\tau }_{0}\right)$$

In order to obtain this discretized version of the wavelet family, $\left\{\Psi_{j,k}\left(t\right)\right\}$, the relations used were: $s=s_{0}^{j}$ and $\tau =ks_{0}^{j}\tau_{0}$. A common choice for $s_{0}$ is $s_{0}=2$, which leads to the wavelet used in the case of the transform called Diadic Wavelet Transformation. If we now refer to a continuous time signal *x*(*t*), the discretized version of the continuous wavelet transformation will be of the form:

(4) $${\mathrm{DWT}}_{x}\left(j,k\right)=\int_{-\infty }^{\infty }x\left(t\right)\psi_{j,k}^{*}\left(t\right)dt$$

The relationship mentioned above actually defines a scalar product between our signal *x*(*t*) and a function in the family $\left\{\Psi_{j.k}\left(t\right)\right\}$. It resembles the relation that allows the calculation of the Fourier coefficients of a periodic signal. Daubechies [51,52] showed that for there to be a wavelet function $\left\{\Psi \left(t\right)\right\}$, there must be another function called the scaling function, which has the notation *φ*(*t*). The scaled versions of this function are $\emptyset_{j}\left(t\right)=\emptyset \left(2^{-j}t\right)$. Any wavelet function at scale *j* can be generated as a linear combination of scale functions at scale *j* − 1. For example, a wavelet mother wave of scale 0 can be written like this:

(5) $$\psi_{0}\left(t\right)=\sum_{k}a_{k}\varphi \left(2t-k\right)$$

One property that is very well known when talking about wavelet functions is that they can generate orthogonal bases of L^2^(ℜ).

In order to be able to prove that this property is valid, we need for the family $\Psi_{j.k}\left(t\right)$ to satisfy two conditions, namely:

1. The condition of orthogonality
2. To form a complete basis.

For the second condition, it is necessary that any signal in L^2^(ℜ) be written as a linear combination of functions in the wavelet family. This property from a mathematical point of view can be formulated as in the relation below:

(6) $$\langle \psi_{j,k},\psi_{m,n}\rangle =\left\{\begin{array}{c} 1,\;\mathrm{if}\;j=m\;\mathrm{and}\;k=n\\ 0,\;\;\;\;\;\;\;\;\mathrm{otherwise} \end{array}\right.$$

In a particular case that can be highlighted, this property is also valid if we restrict to a single decomposition scale (i.e., *j* = *m* in the above equation). In this situation, it can be said that the family that is obtained by translating in time the mother wave Ψ(*t*), namely:

(7) $${\left\{\Psi \left(t-k\right)\right\}}_{k\in Z}$$

represents an orthonormal family.

### 3.4. EEG Analysis to Determine Drivers’ Drowsiness and Cognitive State

The acquisition of EEG signals for drivers of transport vehicles may be performed using a non-contact capacitive type system. The capacitive sensor is installed in the back of the driver’s seat (headrest), thus monitoring the EEG signals in the occipital area. The principle diagram for a non-contact sensor is shown in Figure 2. The EEG signals that are to be monitored and analyzed, in order to determine the states of fatigue and drowsiness, are:

- Alpha: studies have shown that for an awakened person, the presence of alpha waves indicates relaxation. Alpha waves are in the range of 8 Hz to 12 Hz and have an almost sinusoidal shape and a level between 5 and 150 microvolts [μV] (typically between 20 and 50 μV).
- Beta: When a person thinks or responds to an external stimulus, alpha waves are replaced by beta waves. These are in the range of 14 Hz to 25 Hz and have a lower amplitude.

According to research in the field and our studies [53], we found that alpha and beta waves are associated with open/closed eye movements, and this variation of alpha and beta waves can highlight the installed drowsiness and low attention/concentration to drivers in transport systems.

#### EEG Analysis Using the Welch Method

Aperiodic finite energy signals are analyzed in the frequency domain using the Fourier transform. In order to estimate the spectral characteristics of the signals considered to be random processes, it is not possible to apply the Fourier analysis directly, but a statistical treatment of them is adopted. The Fourier transform of the autocorrelation function of stationary random processes, which represents the power spectral density, makes the connection between time and frequency domain. Based on a finite set of observations, the spectral components of a random process can be extracted. In this article, the random process is represented by brain activity (EEG signal).

To determine the estimated power spectral density, the following nonparametric methods can be used: direct method (periodogram), Bartlett method (mediated periodogram), Welch method (modified mediated periodogram) and Blackman–Tukey method. For the experimentally obtained data, the nonparametric Welch method has been applied.

Next, we make a brief presentation of the mathematical support on which the Welch function algorithm is developed. The segments obtained from the initial vector can overlap, and a window is applied to each segment. Let *x*[*n*] be a sequence of N. The segments are obtained as follows:

(8) $$x_{i}\left[n\right]=x\left[n+iD\right]\;for\;n=0,\;1,\;2,\ldots ..,\;M-1\;and\;i=0,\;1,\;2,\ldots ,\;K-1$$

The results are *K* segments, each of length *M*. If *M* = *D,* the segments do not overlap. If it has the value $\frac{M}{2}$, there is 50% overlap between successive segments and *L* = 2*K* segments. *K* segments of length 2*M* each can be obtained. This overlapping of the segments causes a reduction of dispersion. Before calculating the periodogram, the data segments are weighted with a window, which leads to a modified periodogram:

(9) $${\tilde{P}}_{xx}^{\left(i\right)}\left(f\right)=\frac{1}{MxU}{\left|\sum_{n=0}^{M-1}x_{i}\left[n\right]w\left[n\right]e^{-j2\pi nf}\right|}^{2}\;\;\mathrm{for}\;i=0,\;1\ldots .L-1$$

where *U* is a factor of power normalization of the window function and is chosen as:

(10) $$U=\frac{1}{M}\sum_{n=0}^{M-1}w^{2}\left[n\right]$$

The use of the window function has the effect of reducing the lateral lobes and, therefore, of the spectral leakage phenomenon. The estimated Welch power spectral density is the arithmetic mean of these modified periodograms:

(11) $$P_{xx}^{w}\left(f\right)=\frac{1}{L}\sum_{i=0}^{L-1}{\tilde{P}}_{xx}^{\left(i\right)}\left(f\right)$$

The power spectral density was estimated on 8-s segments of the initial signal. In order to obtain the representation of the recorded signal in the figures below, the following steps were followed:

- acquisition: with a sampling frequency of 1 kHz;
- filtered: Notch filter (50 Hz frequency rejection), bandpass filter (8–12 Hz alpha rate and 14–25 Hz beta wave);
- Welch analysis is performed.

### 3.5. O_2_ Saturation Monitoring

In severe cases, hypoxemia can interfere with brain activity, producing headaches and breath shortness. These symptoms may affect the ability of a non-autonomous car driver to react to different stimuli in the normal driving process. This is why we also consider it necessary to evaluate the combination of blood oxygen saturation and pulse. The SpO_2_ monitor (pulse oximeter) non-invasively measures the concentration of O_2_ in the blood (O_2_ in the blood is bound to hemoglobin, and only a small part is dissolved in the plasma). The principle of operation of the pulse oximeter is based on absorption spectrophotometry (Beer–Lambert law), which measures the changes in light absorption by two forms of hemoglobin (HbO_2_—oxyhemoglobin and Hb-reduced hemoglobin).

**Figure 7:**
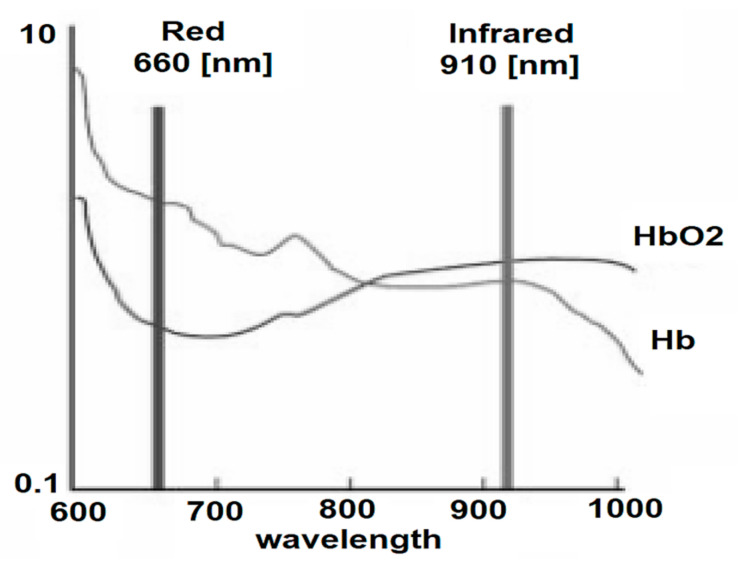
Light absorption by the two forms of Hb.

The pulse oximeter uses two light sources: an infrared spectrum source (IR—910 nm) and a visible–red spectrum source (R—660 nm) and a photoreceptor (PIN diode). The light sources and the photoreceptor (optical sensor) are mounted in a pair that attaches to the fingertip or earlobe. As the background absorption of radiation by venous blood occurs, subcutaneous tissue and skin is practically constant, and the only variable is the amount of Hb (pulsating wave) in the vascular bed [54]. Figure 7 shows the graphs for light absorption by the two forms of Hb.

**Figure 8:**
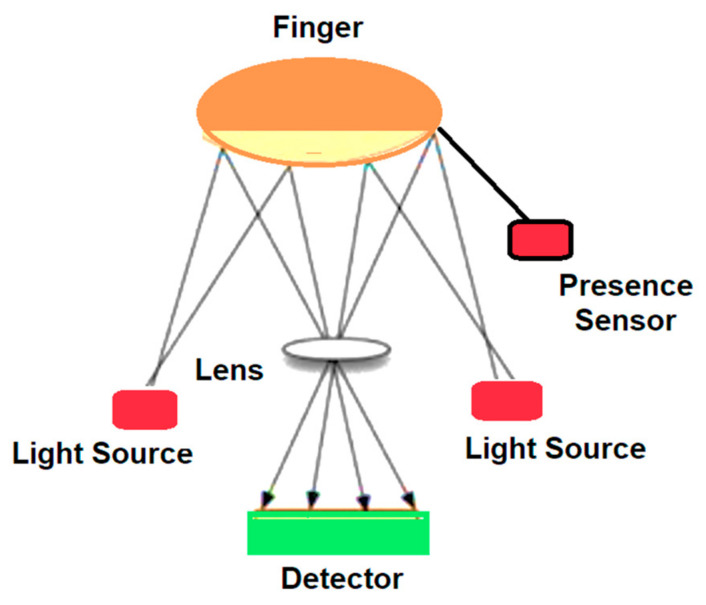
O_2_ saturation sensor modified configuration mounted inside the steering wheel of the vehicle.

Figure 8 shows the configuration of the sensor modified for O_2_ saturation mounted inside the steering wheel of the vehicle.

Oxygen saturation is measured at the top of the pulsating wave to isolate the arterial signal [55,56]:

(12) $$SpO_{2}=\frac{\left[HbO_{2}\right]}{\left[HbO_{2}\right]+\left[Hb\right]}\;\;\left[\%\right]$$

To eliminate the effects produced by venous blood or other tissues, the differences in absorption given by the arterial pulse are measured in relation to the two light sources used according to the formula:

(13) $$R=\frac{\log\left(I_{AC_{R}}/I_{DC_{R}}\right)}{\log\left(I_{AC_{IR}}/I_{DC_{IR}}\right)}$$

where $I_{AC}$ and $I_{DC}$ represent the alternative component, respectively, and the continuous component (of R and IR) of the signal intensity is measured at the photoreceptor.

In practice, the relationship between SpO_2_ and R is not perfectly linear. Therefore, for the correct determination of SpO_2_, the pulse oximeter uses a conversion table (stored in the EEPROM memory of the microcontroller).

### 3.6. Algorithm for Cooperative Driving and Enhanced e-Call Support

**Figure 9:**
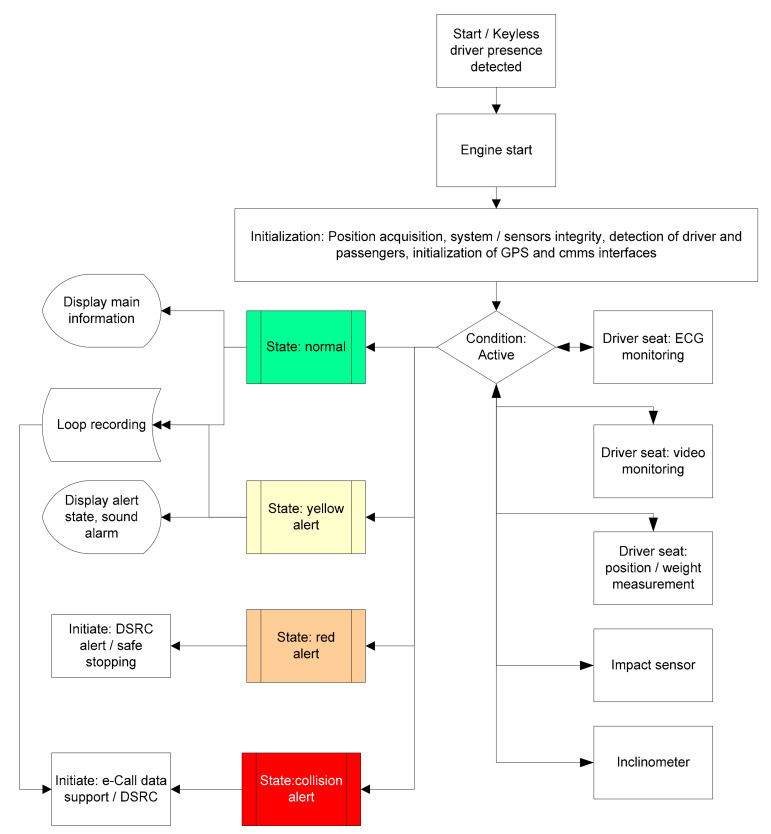
Algorithm for cooperative driving information exchange with enhanced e-Call/telemedicine support.

To increase the overall traffic safety, a solution for merging driver-operated vehicle information with autonomous vehicle information in a cooperative scenario is presented in Figure 9. A description of the algorithm is: the proposed algorithm is intended to be used in a cooperative driving environment (with mixed participants, that is, driver-operated vehicles, semi-autonomous vehicles, and autonomous ones) and able to communicate with each other via C-V2X, DSRC, or other enabled technology. The purpose is to increase traffic safety and to reduce accident rates via an early warning of an abnormal state of a driver, a safe stopping (if available), or a collision alert. Additionally, the algorithm should be able to initiate the enhanced e-Call/other telemedicine-type support, meaning the accident pre-recorded data made available to the rescue team and enabling of the video and sound streaming from the vehicle subjected to the impact. The pre-recorded data may include vehicle ID, number of detected occupants including driver, driver state pre-impact recorded data, dynamic parameters of vehicle before impact, position of the vehicle, and/or other important information that could help the rescue team and shorten the intervention time. It is a well-known fact that seconds matter when a rescue team has to arrive to an accident site to save human lives.

The thresholds between normal, yellow, red, and collision alert may include, but are not limited to (research still in progress):

- Phase 1: system training: the driver’s specific patterns are “learned” by the system during a pre-determined period or via an assisted process at initial configuration. Alerting: initial configuration and system calibration.
- Phase 2: normal state (green) when average recorded parameters are in a pre-determined threshold and no abrupt variations are recorded or metrics values exceeding certain thresholds constantly (e.g., pulse rate: 70–90, oximetry 100–95, etc.). No alerting is performed.
- Phase 3: alert state (yellow) when a single metric exceeds over a declared period of time (e.g., pulse rate over 100, oximetry 94–90). Alerting modes: sounds and displaying of abnormal values. The system may require specific acknowledgement measures, such as reducing speed or pressing a button, according to safety rules.
- Phase 4: alert state (red) when at least two metrics exceed maximal threshold values (e.g., pulse rate between 40–60 or 100–120, oximetry below 90, ECG detects cardiac arrhythmias, EEG detects drowsiness state). Alerting modes: sound, display, reducing speed of car or performing automatic safety stopping for autonomous cars. In case of connected vehicles: alerting neighboring vehicles or vehicles behind.
- Phase 5: collision alert. This phase occurs only in case of a collision, detected by the impact and position/inclination sensors (not part of the solution developed here). In this case, pre-recorded or actual medical parameters are made available for e-Call or telemedicine services.

## 4. Results

### 4.1. IoT System for Data Acquisition

This system is based on the Bitalino multi-sensor acquisition system and combines several sensors developed in research for the acquisition of EEG and ECG signals and MEMS sensors capable of measuring temperature and locating car components that may have temperature increases. Internet access is provided by the use of the ESP8266 microcontroller, which can be installed in the on-board computer of the vehicle, and the mode of interaction with the system in the vehicle is achieved by using a display system. The system is made of several interconnected subsystems and modular components to have high adaptability and increased efficiency, using all resources.

**Figure 10:**
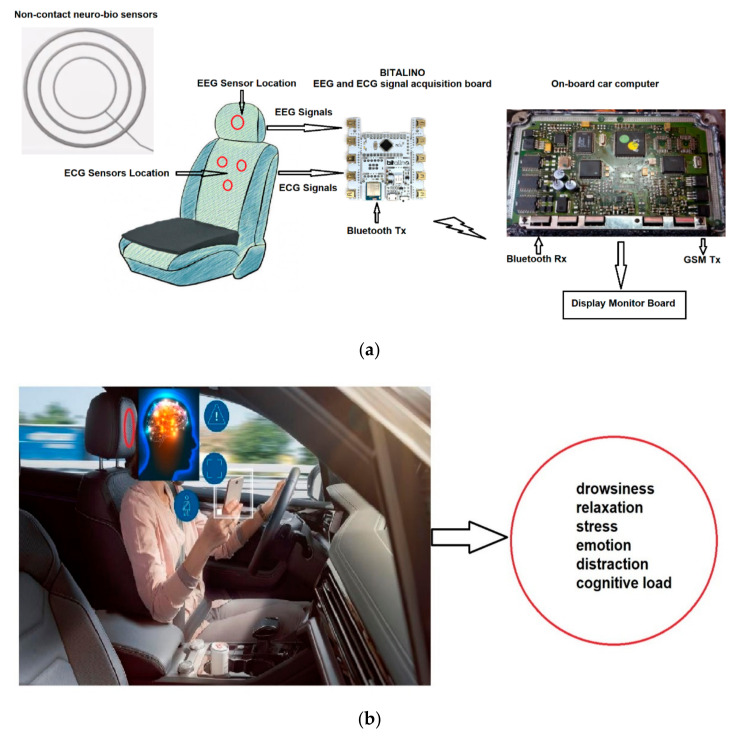
(**a**) IoT system block diagram for the acquisition of biomedical information; (**b**) shows the location of the EEG non-contact sensor in the head restraint of the driver/passenger seat, which acquires the cerebral signals from a distance of max 10 cm and the analyzed cognitive states.

Figure 10a shows the block diagram of the system, which has the following components: EEG and ECG sensors developed in the project and the modality to install them in the headrest and backrest of the car, Bitalino multisensor acquisition board, and data acquisition by the on-board computer and their transmission to the e-Call/telemedicine system using the ESP8266 microcontroller. In tests, a laptop was used instead of the car’s on-board computer.

Figure 10b shows the location of the EEG non-contact sensor in the headrest of the driver’s/passenger seat, which acquires brain signals from a distance of max 10 cm and cognitive states that can be analyzed (drowsiness, relaxation, stress, emotion, distraction, and cognitive load) using the proposed system.

**Figure 11:**
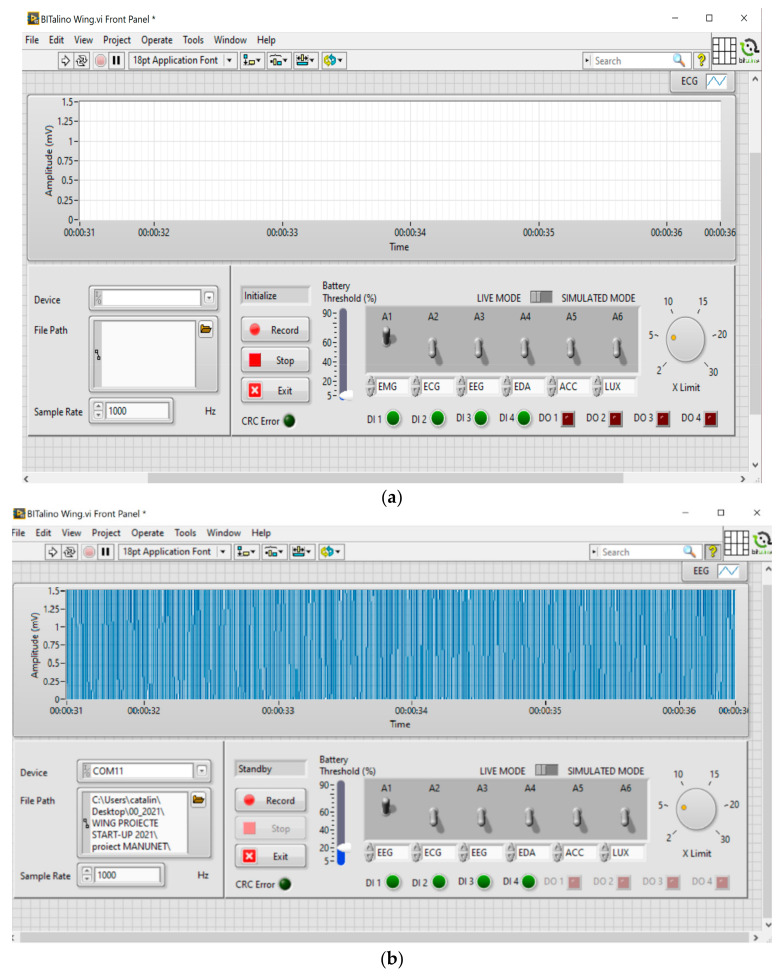
(**a**) GUI Software application developed for the acquisition of biomedical signals using Bitalino hardware; (**b**) the acquisition of EEG signals from the driver/passenger is presented.

Figure 11a shows the user interface (GUI) developed in the project for the acquisition of signals from sensors through the Bitalino acquisition system, and Figure 11b shows the acquisition of EEG signals from the driver.

Next, we present the results obtained for the processing and analysis of biomedical signals.

### 4.2. The Results Obtained for the Processing of ECG Signals

As the capacitive systems installed inside the vehicles are composed of hardware and software elements, and the algorithms for processing the received ECG signals are installed in the monitoring centers (telemedicine), the algorithms for filtering, processing, and analyzing the ECG signals become very important.

**Figure 12:**
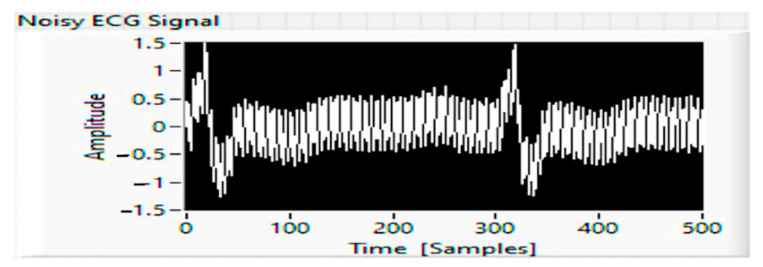
Acquired ECG signal with noise.

Materials and methods: The experiments with the capacitive ECG sensor were performed in the university laboratory, with the captive sensor powered by batteries. The results of the obtained ECG waveform are shown in Figure 12, and the measured values are shown in Table 1.

**Figure 13:**
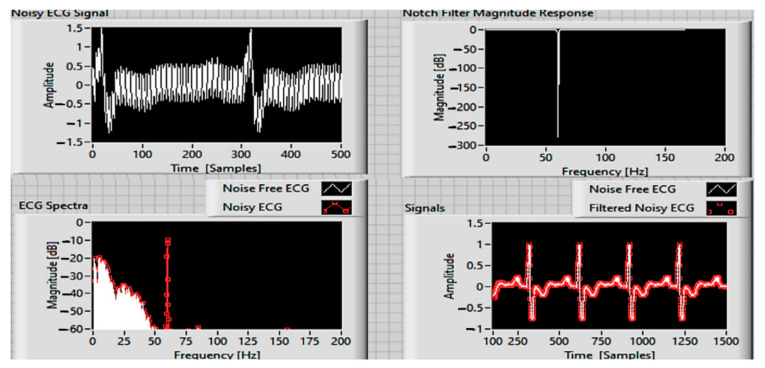
ECG signal filtering results in the 0–35 Hz band of interest.

The software results obtained for a medium sliding filter, followed by a Butterworth filter, in band-pass configuration are shown in Figure 13, where frequency spectrum and magnitude response show that 0–35 Hz is the band of interest.

Wavelet transformed ECG signal analysis is an optimal method because it does not require large resources in terms of hardware and software. For autonomous vehicles, this is a very important aspect, as these types of applications should not consume too much of the information processing resources of such a vehicle, which are already in high demand due to the autonomous driving process. WT processing has the advantage that it retains the shape of the signal, allowing only changes in extension over time.

**Figure 14:**
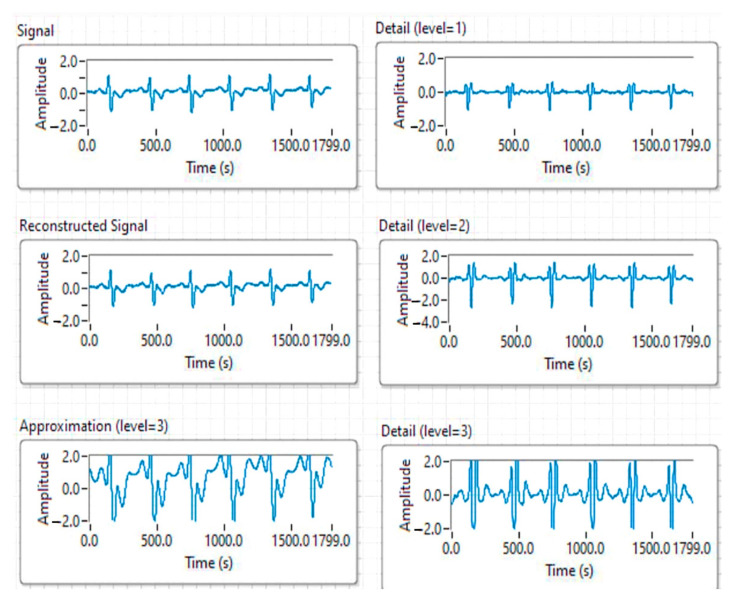
ECG signal analysis results using wavelet transform and Haar function.

Figure 14 shows the good result obtained with the Haar SWT function on three levels; the specific soft values for the threshold were selected after several attempts.

### 4.3. EEG Analysis to Detect Drowsiness and Cognition State

#### 4.3.1. EEG Analysis Using the Welch-Type Power Spectral Density Method for Drowsiness

To estimate/predict the occurrence of drowsiness of drivers, EEG recording was performed using the capacitive sensor installed in the seat headrest. Using the PSD Welch method, the power spectra for alpha, beta brain waves were determined, which show by their succession the closing and opening of the eyes (blinking of the eyes).

**Figure 15:**
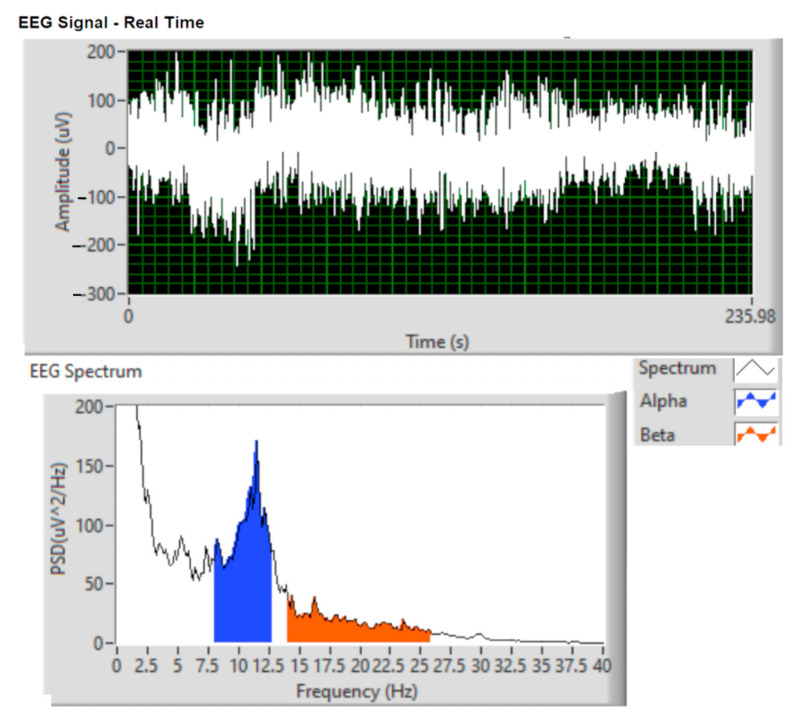
Frequency spectrum for alpha and beta brain waves when eyes are open.

**Figure 16:**
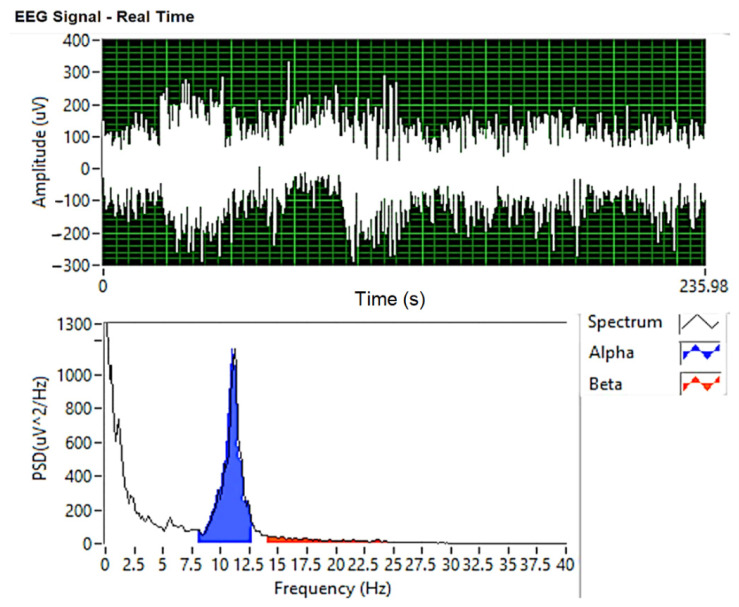
Frequency spectrum for alpha and beta brain waves when eyes are closed.

Figure 15 and Figure 16 show the spectra of alpha and beta brain waves analyzed for attention span and the time of drowsiness.

From the comparison of the spectra represented in Figure 15 and Figure 16, we notice that the amplitude of the alpha waves increased and the beta decreased during the state of relaxation, showing the onset of drowsiness. Following studies and research in the field of EEG, increasing the aptitude of alpha waves is associated with the installation of states of relaxation and drowsiness, and increasing the amplitude of beta waves is associated with thinking and responding to external stimuli.

#### 4.3.2. EEG Analysis for Cognitive State Using the Modified RBF Network

**Figure 17:**
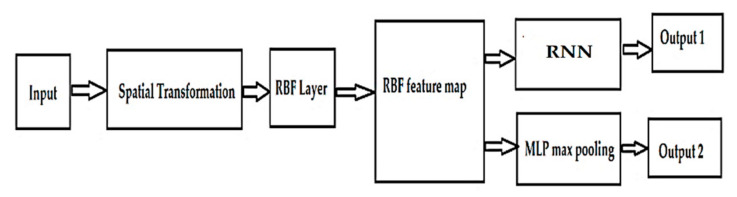
The architecture proposed for a modifed RBF network.

The architecture of the proposed network may be observed in Figure 17. For classification, we proposed a modified RBF network (RBFMod) based on a recurring neural network (RNN) architecture, to which we added an additional MLP layer to increase performance.

A completely different class of networks is recurrent neural networks (NNRs). As the name suggests, this family of networks assumes that information is returned to the network. Recurrent neural networks (NNRs) are specially designed to classify the data that make up the sequences of cognitive states (stress, emotion, relaxation, distraction). The essential difference between the proposed neural network and the classical neural networks is represented by the recurrent layers in which the connections between the neurons are made cyclically. RNNs receive at input a series of elements that belong to a sequence and generate the next element in the sequence—depending on the network architecture—input vectors related to cognitive states, and the values of the parameters resulting from the testing process. Unlike the problems studied so far, recurrent networks specialize in the analysis of data sequences. Each sample in the sequence is analyzed in order, with the result then being returned to the network to participate in the processing of the next sample. An extension of the RNN is the LSTM (Long Short-Term Memory) network, in which the recurring layers have a series of additional components, called gates. These have the role of alleviating the problems caused by gradients with too low or too high values (disappearance gradients, explosion gradients). The proposed modified RBF network contains the following modules: a spatial transformation module for EEG signals, a module for extracting the characteristics of cognitive salts resulting from RBF, and an MLP to combine the signatures of recognized cognitive salts from EEG signals in the vicinity of each nucleus. By using spatial transformation, the input samples will be aligned in a canonical space so that they are invariant to the artifacts that can influence the stability of the signals and, implicitly, the recognition and classification of cognitive states.

**Figure 18:**
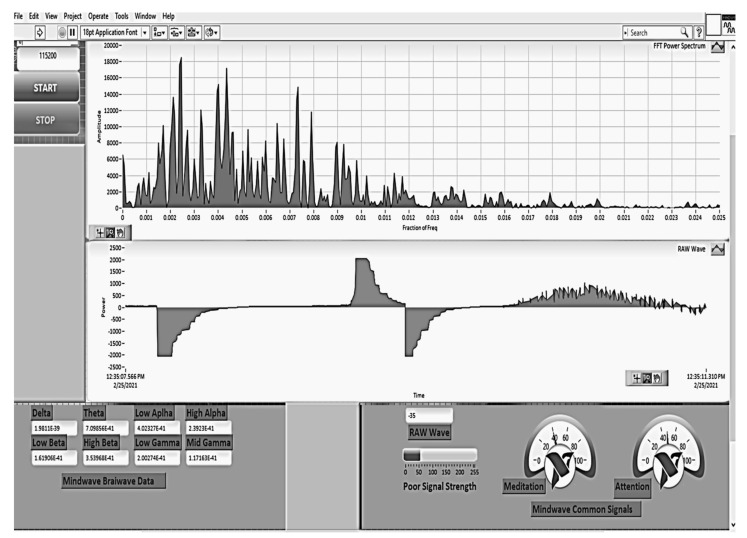
Simultaneous detection of cognitive states of meditation and attention using the modified RBF neural network.

Figure 18 shows the results of simulated detection of cognitive states of meditation and attention using the modified RBF neural network.

### 4.4. Simultaneous Multiple-Point Measurement of Temperature Based on a MEMS Technology Sensor

To determine the temperature changes inside the vehicle, or to determine the heating/overheating of certain components in the construction of vehicles and to prevent the occurrence of flames, a MEMS sensor type AMG8834 was used. This sensor is based on the analysis of infrared radiation (IR) of the environment inside the vehicle, making it possible to scan it based on 64 measuring points, passively, thus allowing monitoring areas of interest, setting the temperature gradient, and imposing prayers of alarm.

The sensor has the ability to measure the temperature of a surface (human body, car parts) at several points and not the ambient air temperature, as existing modules on the market. It mainly targets the use of the system inside autonomous vehicles; as they become more and more advanced, they will act as a driver, taking children from school, transporting injured people as an ambulance, and many other activities that will become common in everyday life. As there will be no person in the car to monitor, the need for an alert in case of danger is growing. Vehicles usually heat up quickly, with much of the indoor temperature rising in the first 15 to 30 min; indoor temperatures rise very quickly and have been found to reach a critical temperature of 40 °C in about 8 min on a summer day. The system will be conveniently placed inside the vehicle.

**Figure 19:**
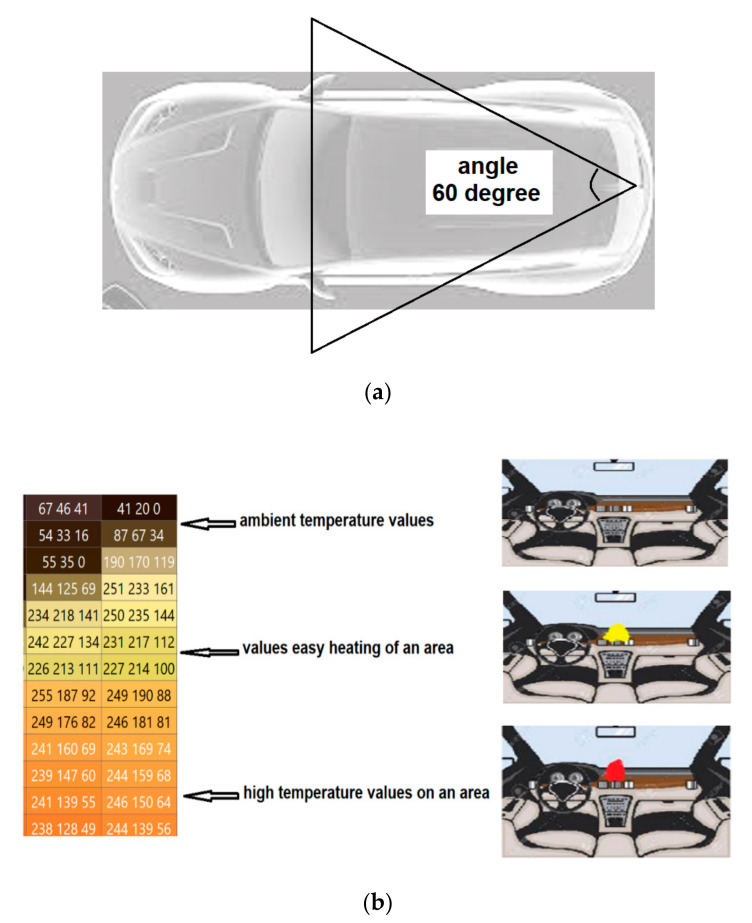
IoT temperature measurement system. (**a**) MEMS sensor location and field of view; (**b**) changing pixel values for ambient temperature (black), values after light heating (yellow), and high flame temperatures (orange).

For this, the best location for the sensor was studied in order to determine its optimal position so that it will have the largest field of view. Based on this information, it was chosen to place the sensor in the rear of the vehicle in order to have a field of view as large as possible, so that it could detect infrared radiation coming from both the rear seats, but also from the front and from the dashboard. Figure 19a shows the location inside the vehicle of the temperature monitoring system with MEMS.

MEMS AMG8834 sensors can be used in a Grid-EYE network, as they are a versatile configuration and economical sensor, being ideal for IoT applications. In this research, we proposed the installation of a Grid-EYE network inside the vehicle to prevent potential overheating and fires. The system was tested and calibrated experimentally by creating different heating temperatures, and the results were measured and compared. High temperatures (flames) were detected and analyzed by the sensor based on changes in pixel values in the successive frames analyzed. Thus, using the infrared temperature detection device, it was possible to passively map the temperature and the thermal distribution for 64 points of a scanned surface without intrusion, which allowed, depending on each scanned area, the establishment of acceptable temperature, as well as alarm thresholds. At the same time, for certain scanned surfaces, the gradient of the temperature change can also be set, as well as the alert thresholds. The proposed system may be the basis for the development of other subsequent applications, such as alarm systems and burglary prevention. In stark contrast to single-element thermal sensors and pyroelectric sensors, the system can simultaneously detect multiple people on the move or in steady position, and surface temperature can be measured extremely accurately in real time.

Figure 19b shows the transition of pixel values for ambient temperature (black), values after a slight warming (yellow), and high flame temperatures (orange). When heating, overheating, or flame temperatures are detected, the MEMS network sends alerts on board the vehicle and on the internet to the data center.

The MEMS Grid-EYE network proposed in the article can also be used for other IoT applications, such as burglary detection systems, because the proposed network can simultaneously detect several moving people, and temperature changes are determined in real time with high accuracy.

### 4.5. Solution for Viewing and Centralizing Online Data Received from IoT Devices

**Figure 20:**
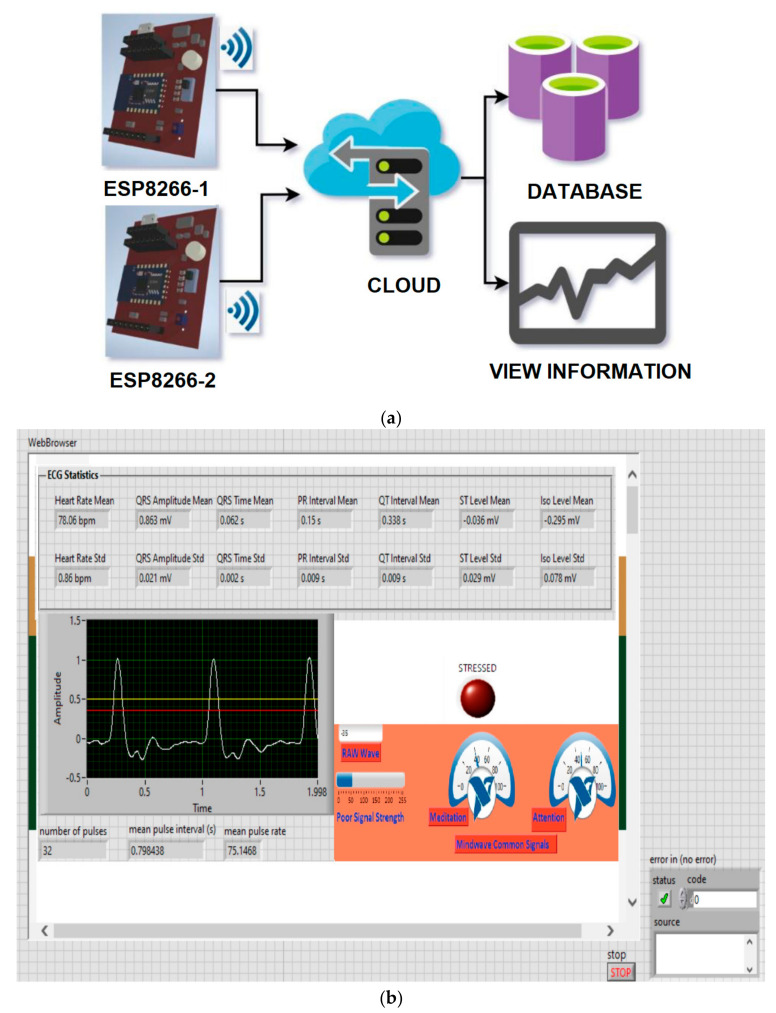
(**a**) IoT management system diagram for a sensor node; (**b**) view of the WEB page developed for data integration.

In order to be able to view and store the data received from the IoT sensor nodes, several existing options were researched. The problems that needed to be solved were the visualization of the data from each sensor node on a map, the storage of data in the cloud for its analysis, the alert signaling of possible real-time events, and the visualization of information received from sensors using the cloud, while also pursuing the security of communications. Several options on the market that offer solutions for managing devices connected to the internet have been studied and some of them have been chosen for extensive analysis. The exclusive use of online platforms without the use of local servers for better data management and security was considered. Some of the important information that needs to be viewed in real time is location data, the time the data were sent, and sensor information. Solutions were also sought for sending alerts on a smartphone device in case of possible dangers. The system needs a node with two ESP8266 microcontrollers mounted inside an autonomous vehicle, one of them for monitoring the temperature levels of surfaces or people in the passenger compartment. It needs also to alert, using the application installed on the smartphone, the user of the presence of people or animals inside, which may be in an alert situation due to an excessive temperature in the passenger compartment or by monitoring the cognitive condition and heart rate of the passengers in the vehicle. The second ESP8266 microcontroller present in the passenger compartment needs to take care of collecting data from the sensors and transmit it to the cloud to be analyzed and viewed in real time (Figure 20a).

The purpose of the IoT network proposed in this article is to assess the states of drowsiness, stress, and the level of oxygen saturation for the driver of the vehicle and detect, in advance, the possible problems that may occur inside the vehicle or certain components of the car. The system also maps people inside the vehicle, enabling it to issue real-time alarms in the event of potential danger and store information in a database.

To achieve the system of visualization, transmission, and storage of data from IoT nodes installed inside a vehicle or in any means of air, railway, and naval transport, we tried to propose an optimal solution by performing an analysis of the systems currently used. The problems we wanted to solve were the visualization of data from each IoT node related to physiological sensors (EEG, ECG), temperature and oxygen saturation monitoring on a virtual map with a display in a web browser, storing data in the cloud for further analysis and signaling the occurrence of possible events in real time, and achieving the security of data communications. The proposed solution used online platforms without the use of local servers because this architecture ensured good data management and security. Another argument in choosing this solution was that the data viewed in real time were given by location, time of occurrence of events, and physiological information obtained from IoT sensors. The database could be transmitted to a dispatcher (security service, car insurance, emergency e-Call system 112) in case of accidents so as to reach a high level of information on the condition/health of passengers in time.

Regarding our research, after the implementation of the IoT architecture of the proposed system, the next step was to develop a web page, using Google Sites, to facilitate access to centralized data visualization. The web page was made using HTML, CSS, and JavaScript, and Figure 20 shows some of the information available on the web browser page. The web browser application was made in LabVIEW.

For further developments, we are looking for solutions to expand the IoT network related to physiological sensors for all people inside the vehicle. This proposal can be used for future autonomous vehicles/drones. The architecture we will use to expand the IoT network involves using a server and an application installed on a smartphone device, so that the IoT network user can view the acquired data in real time and receive alerts in case of potential events that occur. The ultimate goal is the management of all devices by a single operator.

To compare the results obtained with the proposed method, we referred to the following articles [57,58,59,60,61], which focused on the automatic detection of drowsiness and cognitive states for the driver of a vehicle. The comparative results are presented in Table 2.

## 5. Discussion and Conclusions

In this article, a proposal on creating a multi-sensory system for detecting/estimating drowsiness, ECG parameters, and stress levels for non-, semi-, or fully automated cars’ drivers has been investigated and tested in laboratory conditions. To this ensemble, a module for predicting the possibility of overheating vehicle components has been added. The purpose was to improve the response speed, efficiency, and effectiveness of emergency services for all types of cars equipped with this sensing infrastructure. The analysis of biomedical signals performed by the system was based on the analysis of the power spectrum density (PSD) of the electroencephalogram (EEG) and the correlation with the heart rate (ECG), or the respiratory rate. In this project, it was proposed to collect EEG and ECG data, using a sensory system of a capacitive type installed in the headrest and the back of the driver’s seat, which does not involve the wearing of sensors by humans. Oximetry was also added with the intention of helping to more effectively assess the consciousness and healthy state of different occupants of a vehicle, mostly for the person occupying the driver’s seat. With the help of this screening, analysis, and diagnosis system, a significant increase of both safety and security in the field of road transport is expected to be achieved. The solution addresses the most frequent causes of road accidents: human attention and ability to react. Additionally, the presented system can be connected to an emergency system (such as e-Call), and the physiological parameters can be transmitted to the emergency medical team. Despite the efforts made by the research team and collaborators, we consider that there is still a a considerable amount of work to perform in this direction in order to produce a finite, expandable, and reliable solution that is ready for inclusion in the majority of vehicles. This is due to the numerous factors that may influence the precision and trust of indirect measurements of human health and drowsiness metrics. Therefore, we consider that our proposed solution is just a starting point for future investigations that must expand this system for the provision of more accurate medical care information (telemedicine/telemonitoring) remotely for patients with heart problems and cognition. Among the people targeted, an important category which should be prioritized, are drivers with different types of disabilities.

In conclusion, the article presents an IoT system for monitoring the physiological states (cognitive, ECG) of passengers, pulse oximeter, and monitoring the possibility of heating of some components of the structure of a semi-autonomous/autonomous vehicle. The novelty is determined by the development and placement of contactless sensors for the acquisition/analysis of EEG/ECG signals that are located in the headrest and back of the driver’s/passenger seat, acquiring biomedical signals from a maximum distance of 10 cm and also by algorithms implemented for analyzing them. As can be seen from Table 2, the novelty of our proposal consists primarily of the use of an EEG sensor without contact with the human subject, because the methods presented in the literature use an EEG headset, and the detection is performed with 96.75%.

Additionally, the proposed system can be installed in any semi-autonomous and autonomous car, allowing the transmission of acquired data on an IoT structure to the e-Call system or telemedicine systems.

An important aspect for the practical application of the proposed device and method is related to the connection made with the emergency medical system and with the mobile telephony systems to improve telemedicine services.

Real-time telemonitoring of patients, both preventively and after major medical events, is a procedure increasingly used in medical practice. It requires devices with increasing performance for the acquisition and transmission of vital parameters. A limitation of telemonitoring of vital parameters is the failure of these systems to detect important physiological changes: the detection of massive blood loss, insufficient plasma volume in patients with burns, or the identification of serious diseases in children. Vital parameters within normal limits do not guarantee a stable physiological state, thus suggesting that the usefulness of their telemonitoring is rather an indicator of the need for more future investigations. The telemonitoring systems shown can be used as alarm systems in case of monitoring during normal activity or physical exercise. The effects of the large-scale introduction of telemonitoring systems are materialized by increasing the access to modern technologies in the medical field and the quality of the medical act, by reducing equipment costs, having as result an increase in population’s health, thus a decrease of mortality. From their point of view, patients can save time, money, and comfort by maintaining or increasing the quality of the medical act, physically lowered right at the patient’s home. From an economic point of view, the introduction of telemonitoring systems achieves a reduction in social costs in the field of health, not infrequently quite expensive. The costs of acquiring, storing, and managing medical data by automating them will be significantly reduced. Continuous telemonitoring of patients will reduce hospitalization periods and patient time in polyclinics and medical practices. Telemedicine systems will gradually complement the classic medical data storage systems, which currently take up a lot of space, require significant time for data access, and do not allow the recording of complex data. Finally, it is found that information systems, in the case of telemonitoring based on embedded systems, actively and efficiently compete for the quality of medical decisions, especially in situations where the lack of an in situ specialist is still a reality on the threshold of the third millennium.

## Figures and Tables

**Table 1:** Operating values for the ECG sensor.

Parameter	Voltage [V]	Amplitude Pk-Pk [V]	Load Current [A]	Signal Shape
Value	1.8–3.5	1.82	163 × 10^−6^	good

**Table 2:** Comparison of methods used in the automatic detection of drowsiness and cognitive states.

Reference	Year	EEG Sensor	Method Used	Detection Result
[57]	2021	EEG headset	CNN	94.68%
[58]	2021	EEG headset	JTFA	92.00%
[59]	2020	EEG headset	MLP	95.35%
[60]	2020	EEG headset	MLP-CNN	96.50%
[61]	2019	EEG headset	PCANet	95.00%
Proposed Method	2021	EEG non-contact	RBF Modified	96.75%

## Data Availability

The data used were obtained in the laboratory of Intelligent Transport Systems within the Faculty of Transport, Polytechnic University of Bucharest. The data obtained are not public.
